# The Scope and Impact of Viral Infections in Common Variable Immunodeficiency (CVID) and CVID-like Disorders: A Literature Review

**DOI:** 10.3390/jcm13061717

**Published:** 2024-03-16

**Authors:** Adam Al-Hakim, Mark Kacar, Sinisa Savic

**Affiliations:** 1 Department of Clinical Immunology and Allergy, Leeds Teaching Hospitals NHS Trust, Leeds LS9 7TF, UK; mark.kacar@nhs.net (M.K.); s.savic@leeds.ac.uk (S.S.); 2 Leeds Institute of Rheumatic and Musculoskeletal Medicine (LIRMM), School of Medicine, University of Leeds, Chapel Allerton Hospital, Chapeltown Road, Leeds LS7 4SA, UK; 3 Allergy and Clinical Immunology Unit, University Clinic Golnik, 36 Golnik, 4204 Golnik, Slovenia; 4 Faculty of Medicine, University of Ljubljana, Vrazov Trg 2, 1000 Ljubljana, Slovenia

**Keywords:** CVID, common variable immunodeficiency, monogenic CVID, viral infections, COVID-19, chronic norovirus, oncoviruses, viral hepatitis, viral gastroenteritis, *NFKB1*, *NFKB2*

## Abstract

Common Variable Immunodeficiency (CVID) is a heterogeneous primary immunodeficiency disorder characterised by impaired antibody production, leading to recurrent infections and an increased susceptibility to viral pathogens. This literature review aims to provide a comprehensive overview of CVID’s relationship with viral infections, encompassing disease pathogenesis, key presenting features, specific monogenic susceptibilities, the impact of COVID-19, and existing treatment options. The pathogenesis of CVID involves complex immunological dysregulation, including defects in B cell development, antibody class switching, and plasma cell differentiation. These abnormalities contribute to an impaired humoral immune response against viral agents, predisposing individuals with CVID to a broad range of viral infections. Genetic factors play a prominent role in CVID, and monogenic drivers of CVID-like disease are increasingly identified through advanced genomic studies. Some monogenic causes of the CVID-like phenotype appear to cause specific viral susceptibilities, and these are explored in the review. The emergence of the COVID-19 pandemic highlighted CVID patients’ heightened predisposition to severe outcomes with viral infections. This review explores the clinical manifestations, outcomes, and potential therapeutic approaches for COVID-19 in CVID patients. It assesses the efficacy of prophylactic measures for COVID-19, including vaccination and immunoglobulin replacement therapy, as well as trialled therapies.

## 1. Introduction

### 1.1. Common Variable Immunodeficiency

Common variable immunodeficiency (CVID) is the most common symptomatic primary immunodeficiency (PID), representing a heterogeneous cohort of patients with hypogammaglobulinaemia of unknown aetiology [1,2]. Though first reported in 1954, the term CVID was coined in 1971, to describe syndromes of immunodeficiency and hypogammaglobulinaemia separate from those with more specific clinical features and Mendelian patterns of inheritance [3,4]. Today, there is a set of diagnostic criteria for the diagnosis of CVID (Table 1), though the disease can generally be characterised by hypogammaglobulinaemia, impaired functional antibody responses, and recurrent infections, particularly affecting the sinopulmonary system [5,6,7]. In addition to the increased susceptibility to infections, individuals with CVID have an increased risk of autoimmune disease, lymphoproliferative disorders, malignancies, inflammatory gastrointestinal complications, liver disease, interstitial lung disease and granulomatous disease [5,8].

CVID has a prevalence between 1/10,000 and 1/100,000, with approximately 1/3 experiencing disease onset before the age of 10 years [8,9,10]. There is a bimodal age distribution, showing peaks in the first and third decades, with a higher incidence in males (2:1) before 11 years and a higher incidence in females (1.3:1) after 30 years [10,11,12]. Inheritance of CVID is suggested by the apparent relationship between CVID and selective IgA deficiency (SIgAD), the most common (often asymptomatic) primary immunodeficiency, in families. Both conditions are seen to occur in different members of the same family, where familial inheritance of either condition is around 20%, whilst SIgAD can develop into CVID [13]. Furthermore, in families where there is autosomal dominant inheritance of CVID/SIgAD, CVID is often seen in the parents whilst SIgAD was more frequently observed in the descendants, suggesting a spectrum of phenotypes arising from the same genetic background [13,14].

Historically, only 2–10% of CVID cases have had a recognised monogenic aetiology, with the rest either undiscovered or driven by oligogenic/polygenic mechanisms [15,16,17]. However, the advent and increased availability of more sophisticated genetic analysis has accelerated the discovery of new rare genetic variants, with recent papers identifying a monogenic cause in approximately 20–50% of CVID cases [18,19,20]. Genes that have been associated with monogenic CVID include *ICOS*, *NFKB1*, *NFKB2*, *IKZF1* (*IKAROS*), *TNFSF12* (TWEAK), *TNFRSF13B* (TACI), *TNFRSF13C* (BAFF-R), *CTLA4*, *CD19*, *CD81*, *CR2* (CD21), *MS4A1* (CD20), *TNFRSF7* (CD27), *IL21*, *IL21R*, *LRBA*, *PRKCD*, *PLCG2*, *PIK3CD*, *PIK3R1*, *VAV1*, *RAC2*, *BLK*, and *IRF2BP2* though diseases related to these are now considered distinct clinical entities, as in the case of activated Phosphoinositide 3-kinase δ syndrome (APDS) [17,21]. Indeed, monogenic causes of ‘CVID-like’ disorders are now excluded from the established CVID classification [7]. Nonetheless, the term ‘monogenic CVID’ is still used contemporaneously to denote CVID-like phenotypes driven by recognised deleterious monogenic variants, and retains validity as most CVID and CVID-like patients are indistinguishable in the clinical setting [20]. As this article is intended for a general immunological audience, ‘monogenic CVID’ remains a helpful way of characterising an important cohort in need of targeted genetic studies, and thus, will be used throughout this article. It is worth noting that, due to these advances in genetic analysis, data from publications before the last 20 years may mention cases of CVID that would more recently be recognised as a form of monogenic CVID, and therefore a separate disease entirely.

### 1.2. Pathogenesis of CVID

CVID is defined in part by hypogammaglobulinaemia, with 85–94% of cases across two large cohorts found to have IgG levels of less than 4.5 g/L, whilst reduced IgA and IgM levels commonly featured [6,11,22,23]. Thus, loss of B-cell function is a unifying factor, driven either by intrinsic B-cell dysfunction or abnormalities in cells which ordinarily stimulate B-cells, disrupting differentiation into switched memory B cells and plasma cells, impairing antibody production, and leading to increased susceptibility to infections [24]. In most cases, there is a reduction in class switched memory B-cells and reduced plasma cells in both the mucosa and bone marrow [25,26,27].

In monogenic CVID, single gene mutations may cause specific disruption of B-cell development or function, affecting isotype switching, somatic hypermutation, or B-cell survival [8,28,29,30]. Recognition of the mutation allows for further characterisation of the affected protein function and related pathways, and in some instances, targeted therapies to arrest B cell dysregulation [24]. In true CVID, with no identifiable causative mutations, there can be a wide heterogeneity of immunological and clinical characteristics, making the exact pathogenesis difficult to determine. Overall, a wide variety of immunological defects have been identified, including irregularities of T-cell numbers/function, Toll-like receptor (TLR) signalling, innate immune responses, dendritic cell (DC) function, thymic maturation and natural killer (NK) cell deficiencies [31,32,33,34,35,36,37,38]. Due to the spectrum of disease and varying underlying molecular basis, it can be difficult to say which of these are driving immune dysfunction and which are a product of the initial abnormality. Though B cell compartments are primarily utilised in CVID classification schemes, studies show a variety of T cell defects are present in around one third of cases [24,26,39,40]. Furthermore, some T cell subset patterns are associated with clinical manifestations, including autoimmunity, splenomegaly, and polyclonal lymphoproliferation [24,39].

Due to its wide heterogeneity, there are increasing efforts to classify CVID cases into clinical subgroups as determined by the immunophenotypic profile. For now, the EUROclass trial represents the current hallmark of classification [26]. In this study, a population of 303 European patients were analysed and their B-cell immunophenotypic markers were used to classify them into cohorts characterised by splenomegaly, lymphadenopathy, granuloma, and autoimmune cytopenias. These findings highlighted the role of dysregulated B-cell homeostasis in disease pathogenicity. More recent studies have removed monogenic CVID from their analyses and demonstrated signature B and T lymphocyte subset profiles, associated with different phenotypes, including infections only, autoimmune and a gastrointestinal/chronic enteropathy [24]. Various forms of monogenic CVID have also been investigated for lymphocyte subset profiles, and, if signature patterns are established, these could provide excellent indicators for further genetic testing in patients presenting with a CVID-like disorder [20,41,42].

Over half of CVID patients—the so-called ‘CVID+’ cohort—suffer from inflammatory and/or autoimmune complications, with autoimmunity alone reported in around one quarter of individuals [43,44,45]. Regarding autoimmune complications, the role of regulatory immune cells, including CD4 regulatory T cells (CD4 Tregs) and the more newly described CD8 regulatory T cells (CD8 Tregs) and B regulatory cells (Bregs) and T follicular helper regulatory T cells (T_FR_), have become targets for investigation [43,44,46]. There is a known association between CD4 Tregs, peripheral tolerance and autoimmune disease, so it stands to reason that dysfunction of regulatory processes in CVID might be driving its autoimmune phenomena. Though clear abnormalities have been demonstrated in these various lymphocyte numbers in CVID, functional analysis is scarce, and typically no consistent association with subset patterns and autoimmune disease is shown, though more work is required [43].

Investigations into the CVID+ phenotype have suggested commensal bacteria and microbial translocation could be driving a state of immune activation, contributing to inflammatory and autoimmune complications [45]. Indeed, there is evidence of increased markers of bacterial infection, less diverse microbiomes, and significantly raised serum bacterial 16S rDNA levels, in CVID+ patients [45]. These results speak to the complexity CVID, where disease manifestations potentially arise as an indirect result of the underlying immunodeficiency, and dysfunctional host-pathogen mechanisms.

Individuals with CVID have a high recognised prevalence of lymphoproliferative disease (17–26%), lymphoma (2–8%), and increased incidence of malignancies including skin cancers, gastric cancers, and leukaemia [47,48]. Proposed carcinogenetic drivers for this phenomena can be split into cell-intrinsic mechanisms, where dysregulated cellular homeostasis, function or cell-to-cell interaction drives cancerous change; and cell-extrinsic mechanisms, where inadequate immune responses allow chronic infection (e.g., oncoviruses), deficient tumour surveillance or dysbiosis, predisposing to malignancy [48].

### 1.3. Common Infective Manifestations in CVID

Infection burden represents a major issue for patients with CVID, even once treated with either prophylactic antibiotics or immunoglobulin replacement therapy (IgRT) [49]. The most commonly observed infections at diagnosis in a cohort of 224 CVID patients were acute bronchitis (62.9%), pneumonia (49%), acute otitis (38.4%), chronic bronchitis (33.9%), chronic sinusitis (36.6%), urinary infections (7.1%), cutaneous abscesses (2.2%), septic arthritis (2.3%), candidiasis (2.3%), bacterial meningitis (1.3%), and sepsis (0.9%) [6]. In this study, the mean age at diagnosis was 26.6 years, immunoglobulin therapy was commenced within 1 year of diagnosis in ~90%, and they were followed up for 11 years on average. During both diagnosis and follow-up the prevalence of any infection, either acute or chronic, was significant, at 87.5% and 86.6%, respectively, emphasising the high infective burden of these patients [6].

A French prospective study of the incidence of infections over 3 years in 252 CVID patients observed bronchitis (69%), sinusitis (63%), pneumonia (58%), gastrointestinal tract infections (27%), meningitis (8%), recurrent shingles (11%), invasive human papilloma virus (HPV) or profuse warts (5%), and severe chickenpox (4%) [49]. In a separate review of 248 patients, notable viral infections reported included viral hepatitis (6.5%) and severe shingles (3.6%) [11].

### 1.4. CVID and Viral Immunity

As stated, CVID is primarily a humoral deficiency, though cellular immunity can also be affected: in one study, 40% of cases were found to have reduced T-cell responses to mitogens and 20% of cases were found to have low CD4 counts [23]. Innate immune function within CVID, such as viral recognition by pattern recognition receptors (PRR) and production of anti-viral cytokines, especially type I (IFN-α and IFN-β) and Type III interferons (IFN-λ), is generally intact [50].

The adaptive antiviral immune response is primarily driven through recognition and destruction of host cells by cytotoxic T lymphocytes (CTL) and NKs, through MHC Class I molecules and specific NK receptors, respectively [51]. CTLs are classically CD8+ though it is increasingly understood that in acute and chronic viral infection CD4+ T-cell subsets with cytotoxic functions are generated and can even replace CD8+ T-cells in chronic viral infection [51]. CD4+ T-cells are classically associated with T helper (Th) cell subsets, though naïve CD4+ T-cells can differentiate into other effector cell subsets, depending on stimulation from antigen presenting cells (APC) and other factors [51]. Th1 T-cells are produced in the context of viral infections and drive the production of antiviral cytokines, promoting cytotoxicity [51]. The importance of cellular immunity and viral elimination is well demonstrated in severe combined immunodeficiency (SCID), a condition defined by an absence of T-cells, where severe and sustained viral infections are a prominent feature [52,53]. Similarly, severe genital herpes is seen in HIV-infected individuals with impaired herpes simplex virus-specific CD8+ CTL responses [54].

Though less central, the role of humoral immunity is elegantly demonstrated by the effectiveness of vaccines in preventing viral disease, achieved through the rapid production of neutralising antibodies to the circulating virus, whilst virus-infected host cells may be recognised and destroyed through antibody dependent cellular cytotoxicity (ADCC) by NK cells and cytotoxic T-cells (CTL) [51]. In addition to producing antibodies, B-cells also modulate the immune response through cytokine production and can act as APC to T-cells via MHC class II molecules, meaning they play a key role in anti-viral responses through various means [51]. Thus, as in the case of CVID or X-linked agammaglobulinaemia (XLA), B-cell dysfunction leads to recurrent and persistent viral infections [37,49].

In addition to this, certain monogenic causes of the CVID phenotype, such as those affecting *NFKB1*, *NFKB2*, *TNFSF12* (TWEAK), *TNFRSF13B* (TACI) and *IKZF1* (IKAROS), have been individually reported with pronounced viral illnesses [55,56,57,58,59]. Some of these genes are known to produce proteins that play a key role in PRR signalling (NFkB family) or CTL and NK cytotoxicity (TWEAK), whilst their dysfunction can lead to anti-cytokine antibodies, or dysfunction of cytotoxic mechanisms, emphasising the spectrum of vulnerabilities in the CVID cohort and the benefit of assessing specific immunophenotypes [51,58,60].

## 2. Methods

The literature review employed a methodical approach to search for relevant and high-quality literature. Four academic databases—PubMed, Embase, Scopus, and Cochrane Library—were systematically searched due to their extensive coverage of biomedical literature. Specific search terms and keywords were selected to capture relevant literature on Common Variable Immunodeficiency (CVID) and viral infections. Boolean operators (AND, OR), truncations (*), and wildcards (?/#) were used effectively to combine search terms. Clear inclusion and exclusion criteria were defined, and the search encompassed research across all publication dates. Inclusion criteria included publications written in English, relating to humans, reports involving CVID patients, and those specifically addressing viral infections and their impact. Exclusion criteria included non-English language articles, articles relating to animals, studies not relevant viral infections or CVID, or those lacking sufficient detail. In addition to database searches, manual hand-searching of article references was performed to identify additional resources. This methodical approach ensured a comprehensive literature search for academic papers.

## 3. Respiratory Viral Infections

Respiratory viruses are common in PIDs, though in CVID they do not typically progress beyond bothersome and regular sinopulmonary infections, causing morbidity through the risk of superadded bacterial infections [61,62]. This is likely due in part to preserved cellular immunity, demonstrated in studies assessing cellular responses to the influenza vaccine, where most CVID patients demonstrated a positive T-cell immune response, indicating at least some degree of preserved anti-viral mechanisms [63,64,65].

A 1998 study demonstrated that in 11 *asymptomatic* patients with CVID on IgRT, with no recent history of infection, 36% (4/11) of patients had evidence of viral infections on samples taken from bronchoscopy, compared to no infections in the three XLA patients and 13 controls [66]. The CVID infections included two patients with adenovirus, one with CMV/adenovirus co-infection and one with adenovirus/rhinovirus co-infection [66]. This suggests that patients with CVID are indeed more susceptible to respiratory viral infections, but that the combination of preserved cellular immunity and IgRT act to prevent active or complicated infections. This was further illustrated by a prospective longitudinal study of 12 patients, 10 with CVID and 2 with XLA, on IgRT followed up for 1 year with a symptom diary, regular viral swabs, and sputum analysis [67]. The 2010 study demonstrated a significantly increased frequency of infections compared to controls, despite adequate immunoglobulin levels [67]. The CVID cohort developed viral infections, including rhinovirus, picornavirus, metapneumovirus, enterovirus, adenovirus, parainfluenza 1 & 2, coronavirus, influenza and respiratory syncytial virus (RSV) [67]. Most viral infections were symptomatic and self-limited, though rhinovirus, which occurred in 80% (8/10) of CVID cases showed a proclivity for recurrent and, often asymptomatic persistent infections [67].

Atypical respiratory viral infections do occur, such as in two cases of CMV pneumonitis, demonstrating a prompt clinical response to intravenous ganciclovir on both occasions [68]. There has also been a reported case of CMV pneumonia in a child with a *NFKB2* loss of function (LOF) mutation [69]. The *NFKB2*-deficient CVID subtype is known to cause autoimmune disease and has previously been associated with both anti-cytokine antibodies and reduced NK cytotoxic activity, possibly heightening viral susceptibility [70,71,72,73]. Similarly, an IKZF1 mutation was associated with severe RSV bronchiolitis in two infants, and one instance of adenoviral pneumonia requiring cidofovir, with immunological studies showing dysregulated T-cell function associated with the disease [57].

These cases collectively show that distinct monogenic drivers, or specific cellular dysfunctions, may predispose to chronic, severe, or atypical viral respiratory infections in CVID. Thus, the clinician should consider performing genetic analysis and cellular function tests on any CVID patient with persisting respiratory infections.

It is well understood that IgRT, the mainstay of CVID treatment, reduces the rate of respiratory infections in patients with CVID, whilst it has been specifically demonstrated to prevent the rate of RSV infections in immunocompromised patients [74,75,76]. Thus, in CVID patients with persisting infections despite antibiotics, optimisation of immunoglobulin therapy, followed by viral screens for consideration of targeted antivirals seem the most rational approaches.

## 4. COVID-19

As of March 2023, when recording stopped, severe acute respiratory syndrome coronavirus 2 (SARS-CoV-2) had caused 6,881,995 deaths worldwide [77,78]. In the UK, there were 24,658,705 confirmed cases and 220,721 deaths, providing a case-fatality ratio (CFR) of 0.90% [77,78]. SARS-CoV-2 is an enveloped, positive-sense, single-stranded (+ss)RNA virus, responsible for Coronavirus Disease-2019 (COVID-19) [79]. COVID-19 has taken a devastating toll across the world, but has proven to be particularly lethal in certain groups, such as the elderly, comorbid, and immunocompromised [80]. Included in these high-risk groups are patients with primary and secondary immunodeficiencies who have frequently been exposed to and infected with the SARS-CoV-2 virus, from which they suffer a significantly higher mortality [81,82,83].

A 2021 UK case series of 100 patients with PID from the early pandemic, prior to vaccinations becoming available, showed a total infection-fatality ratio (IFR) of 17.9%, a CFR of 28.5%, rising to an inpatient mortality of 35.3% [82]. IFR is the proportion of those with presumed COVID-19 who died, whereas CFR represents the risk of mortality in those with a confirmed diagnosis of SARS-CoV-2 infection. This distinction was particularly relevant during the global pandemic, when PCR diagnosis was limited to those requiring hospital review and/or admission. In this same study, outcomes were worse in the 23 CVID patients, with an IFR, CFR, and inpatient mortality of 34.8%, 50% and 61.5%, respectively [82]. This was despite 20/23 (87%) of the CVID patients being on IgRT, the most of any group in the study [82]. For comparison, two studies reporting pre-vaccine COVID-19 outcomes estimated England having an IFR between 1.20–1.57% [84,85].

More broadly, a literature review of COVID-19 outcomes in patients with PID involving 459 patients across 68 studies gave a CFR of 23.5% and inpatient mortality of 34%, confirming the increased risk to patients in this cohort [86]. More specific to CVID, a March 2021 literature review of CVID patients with COVID-19 found 14 publications from countries such as USA (34), Italy (12), France (7), Italy (6), Israel (4), UK (1), Romania (1), Brazil (1), Chile (1), and “unknown” (1) [87]. This review included patients with confirmed serological SARS-CoV-2 diagnosis, both hospitalised and not hospitalised, and found an overall CFR of ~13% between the onset of the pandemic and February 2021 [87].

Following the introduction of vaccines and routine use of therapies, a UK research group later published a case series of 140 UK patients with primary and secondary immunodeficiencies infected between January 2021 and March 2022 [88]. Among those patients, 50 had CVID, with 2 or 3 vaccine doses received in 96% and 86%, respectively [88]. Of this CVID cohort, 10 patients (20%) were hospitalised, and one died, demonstrating an inpatient mortality of 10% and IFR of 2%. For comparison, one study utilised data of 42 million individuals from the national UK census, who were then followed through the National Health Service (NHS) database from March 2021 to March 2022, finding a post vaccine IFR between 0.07–0.50%, depending on the number of vaccines and time from administration of each [89].

### 4.1. Immunological Studies of CVID and COVID-19

Between the use of the vaccines, validated therapies for acute COVID-19 and emergence of the possibly less virulent SARS-CoV-2 Omicron (B.1.1.529) variant, it is difficult to ascertain the exact impact of each parameter individually on the improvement in outcomes in patients with CVID. However, Shields et al. assessed serological responses in 70 CVID patients, with 52.9% responding after the second vaccine and 66.1% responding after the third [90]. Results were reported as an IgGAM ratio to express antibody response to vaccination, with results ≥1.0 considered seropositive [90]. Of all patients with PIDs, individuals with CVID demonstrated the lowest post-vaccine seroprevalence and the lowest median antibody response, highlighting the ongoing risk of morbidity in this population and the importance of other COVID-19 therapies for acute illness [90]. This finding is supported by a study into CD8+ cell responses to the SARS-CoV-2 vaccine in patients with CVID and XLA compared to healthy controls, which showed a greater CD8+ and functional CTL response in XLA compared to controls, but a worse response in CVID compared to controls [91]. This is further supported by studies showing positive specific CD4+ T-cell responses to SARS-CoV-2 proteins in CVID patients, highlighting the role of cellular immunity in their immune response to COVID-19 [92]. Interestingly, this same group demonstrated absent T-cell response in those with severe COVID-19, indicating how dysregulated cellular immunity within the heterogenous CVID cohort may predispose individuals to severe viral infections [92]. A patient with *NFKB2* LOF mutation was reported with severe COVID-19, requiring ventilation, though T-cell studies were mostly unremarkable and he did eventually survive [93].

Accordingly, novel techniques are being developed to cheaply recognise cellular responses to COVID-19, such as the development of an intradermal delayed-type hypersensitivity (DTH) test to SARS-CoV-2 recombinant protein, designed to correlate with ex vivo interferon-gamma release assay [94]. Techniques such as this could conceivably allow for risk stratification of CVID patients in what may eventually become a seasonal disease, and certainly further work is needed in this area of research.

### 4.2. Therapeutic Recommendations for COVID-19 in CVID

Though initially promising, monoclonal antibody therapies for COVID-19 have been increasingly sidelined due to the emergence of new SARS-CoV-2 variants with escape mutations [95]. The antivirals Paxlovid (nirmatrelvir/ritonavir), remdesivir, and molnupiravir continue to be recommended, having significantly reduced hospitalisation and death with early variants and showing benefits in secondary endpoints such as time to recovery with the Omicrom variant [96,97,98]. With regards to supporting humoral immunity, studies of commercial immunoglobulin products have demonstrated substantial levels of anti-SARS-CoV-2 IgG present, sufficient to match values found in convalescent and vaccinated individuals once administered, with one study highlighting a large increase of the antibodies in patient sera post-administration in 35 individuals [99,100,101]. This latter study also demonstrated viral clearance following initiation of IgRT after a median of 20 days in antibody-deficient patients with pre-existing prolonged COVID-19 (median 189 days, maximum > 900 days) [100]. As one might expect due to widespread vaccination and post-infection immunity, the amount of anti-SARS-CoV-2 IgG present in immunoglobulin products appears to be increasing, suggesting increased protection for patients receiving the therapy moving forwards [102].

Regarding these therapies in the CVID cohort, acute, outpatient treatment using oral antivirals or monoclonal antibodies became available after 16 December 2021, through NHS COVID-19 Medicine Delivery Units (CMDU) in the UK. Within the post-vaccination UK cohort, 61.4% (70/114) of eligible PID patients were treated through CMDU, most commonly with the monoclonal antibody sotrovimab (38/70) or the antiviral molnupiravir (16/70) and Paxlovid (10/70) [88]. The study found significantly lower rates of hospitalisation (4.3% vs. 15.9%, *p* = 0.03) amongst individuals treated by CMDU though overall mortality was not significantly affected (2.8% vs. 4.5%, *p* = 0.63), possibly due to study size [88]. These data reinforce the need for early access to antivirals in the CVID population.

In one report, SARS-CoV-2 was associated with acute cerebellitis and myeloradiculitis in a patient with CVID [103]. The patient presented with one week history of difficulty walking and was found to have an acute cerebellar syndrome and myeloradiculopathy in the setting of mild COVID-19 disease. Her neurological function improved following administration of convalescent plasma, steroids, and intravenous immunoglobulin, though it is impossible to draw conclusions on therapy from this case alone [103].

## 5. Mucocutaneous Viral Infections

### 5.1. Human Papillomavirus

The term HPV collectively refers to over 200 strains of non-enveloped, double-stranded (ds)DNA viruses belonging to the Papillomaviridae family [104]. HPV is the most common sexually transmitted infection worldwide and is associated with cutaneous warts, anogenital warts, and a variety of cancers [104]. HPV infection and the development of warts are extremely common and, in immunocompetent populations, these are typically experienced as localised, self-resolving skin lesions that may require a short course of topical therapy to fully remove [104]. However, in immunocompromised patients, especially in certain specific monogenic driven deficiencies, HPV infection and the subsequent warts can be severe, recurrent, and refractory to therapy [105]. Depending on the type of primary immunodeficiency there can be variations in the types, location, and severity of the warts [105].

Regarding CVID, severe warts feature less than in other PIDs, though the French prospective study found 13/252 (5%) of the entire cohort developed invasive HPV or profuse warts, and there are individual reports of severe disease [49,106,107,108]. Notably, in the cases of CVID and severe warts there has tended to be a T-cell impairment, indicating the importance of T-cell function in HPV elimination [105].

One monogenic cause of CVID is TWEAK deficiency, and these cases have been reported as particularly susceptible to HPV [58]. In a report, three family members with histories of recurrent infections, multiple warts, and impaired antibody responses were found to have a LOF mutation affecting *TNFSF12* which encodes TWEAK [58]. TWEAK is known to facilitate cytotoxicity in T-cells and NK cells, with the above study suggesting NK cell dysregulation was responsible for dysregulated local control of HPV [58,109,110]. HPV-associated warts have also been reported in *NFKB2* and IKZF1 monogenic CVID [56,69]. These reports suggest a place for functional immunological testing and genetic analysis in CVID patients presenting with warts.

### 5.2. Herpes Simplex Virus

Herpes simplex virus (HSV) is a highly prevalent enveloped, dsDNA virus, which typically causes oral or genital infections [111]. HSV has two types: HSV-1, traditionally transmitted through oral-to-oral contact and associated with orolabial herpes (cold sores), and HSV-2, transmitted through sexual contact, associated with genital herpes [112]. Both are otherwise known as human herpesvirus (HHV)-1 & HHV-2 respectively, and, along with Varicella Zoster Virus (VZV), belong to the subfamily of alphaherpesvirinae [113,114]. In 2016, it was estimated that 3.7 billion people were seropositive for HSV-1 and 500 million for HSV-2 worldwide [112]. Other manifestations in adults include herpes stromal keratitis (HSK), eczema herpeticum, and meningoencephalitis [111]. Infection is lifelong, with latency occurring in the neurons, though with varying degrees of reactivation and symptom recurrence depending on a number of factors that are not well established [111,115]. Certainly, cellular immunity seems to play a prominent role in HSV suppression, as evidenced by the severity of disease in acquired immunodeficiency syndrome (AIDS) secondary to human immunodeficiency virus (HIV), severe combined immunodeficiency (SCID), and other immunodeficiencies primarily affecting T-cell and NK cell function (e.g., GATA2 deficiency) [54,116,117].

As a disease of primarily dysregulated humoral immunity, CVID is not frequently associated with recurrent cold sores, though CNS and intra-abdominal infections are more frequently reported as discussed further in this article. Nonetheless, there are reports of persisting and recalcitrant oral and genital disease in the population [118,119]. In one such report, a long-standing intraoral ulcer, with HSV positive immunohistochemistry on biopsy, responded only to high dose valaciclovir, having failed to respond to a lower dose a month prior [119]. In another report, an 18-year old NFKB2-deficient CVID patient developed HSV-1 positive herpes vegetans that at first responded to intravenous (IV) foscarnet, before later recurrence and subsequent viraemia, that required a further intravenous course, followed by oral valaciclovir [120]. This patient had reduced NK cytotoxicity on functional assays in addition to in anti-interferon anti-cytokine antibodies (IFN-α and IFN-ω), which correlated with reduced phosphorylation of STAT1 by the same interferons compared to controls [120]. Given the putative blockade of the antiviral interferons and reduced NK cytotoxicity, this perhaps explains the vulnerability to viral infections in this patient, something that has been seen in other *NFKB2* LOF cases [121,122,123]. Recurrent HSV has also been reported in *IKZF1*-associated CVID [56]. These cases highlight the need for diagnosis and surveillance of monogenic CVID subtypes, particularly presenting with recurrent or severe HSV.

#### Treatment of HSV Infection in CVID

Several antiviral drugs are available for the treatment of HSV infection, such as aciclovir, famciclovir, and valaciclovir, which all target the viral DNA polymerase and inhibit viral replication [113,124,125]. These drugs require viral thymidine kinase (TK) for activation, and resistance can develop due to viral mutations affecting TK [126]. Foscarnet, an intravenous DNA polymerase inhibitor that does not require TK for activation, is licenced for aciclovir-resistant mucocutaneous HSV infections in immunocompromised patients [113]. Shared resistance to these agents, which occurs at a greater degree in the immunocompromised population, can result from only a single mutation in the DNA polymerase gene [113,125,126,127]. Two new drugs targeting the helicase-primase complex in both HSV and VZV, and not requiring viral TK for activation, offer a possible solution [128]. Pritelivir and amenamevir seem to show promise as effective drugs in treating acute infection, with the former under clinical trial, though with various promising case reports, and the latter having been approved for use against VZV in Japan [129,130,131]. Pritelivir has been further shown to significantly reduce viral shedding in patients with frequent recurrences, with reports suggesting a much lower incidence of viral resistance, and has been proposed as an option to prevent recurrence in immunocompromised patients [128,132,133].

### 5.3. Varicella Zoster Virus

VZV is another member of the alphaherpesvirinae subfamily, sharing the enveloped linear dsDNA structure [114]. It initially causes chickenpox before latency in sensory ganglia and reactivation as shingles in a dermatomal pattern [114]. There is a relatively high incidence of VZV infection in CVID, with recurrent shingles (11%) and severe chicken pox (4%) both seen in the French study of 252 CVID patients [49]. In one instance, a patient with heterozygous *NFKB2* and AIRE mutations (pathogenicity not stated) and a diagnosis of CVID developed facial VZV infection, trigeminal neuralgia, and meningoencephalitis with viral studies demonstrating vaccine-strained VZV [134]. This report serves to highlight the importance of avoiding live attenuated vaccines in patients with CVID, and how NFKB dysfunction may lead to viral susceptibility, beyond what is seen in CVID as a whole. Other cases of *NFKB2* LOF and severe shingles have been reported [60].

Recently, an inactivated recombinant subunit vaccine, HZ/su (Shingrix^®^), has been developed, showing an excellent vaccine efficacy (97.2%) in immunocompetent subjects over 50 years old [135]. This promises a highly effective prophylactic measure in patients with CVID, in whom the live-attenuated predecessor Zostavax^®^ is contra-indicated. Nonetheless, studies to assess the efficacy of the new vaccine in patients with CVID have not yet been performed, and this represents an avenue for further research before informed recommendations can be made.

### 5.4. Molluscum Contagiosum

Molluscum contagiosum (MC), caused by the dsDNA poxvirus molluscum contagiosum virus (MCV), is a transient contagious dermatosis that commonly affects children, sexually active adults, and the immunocompromised [136], By replicating only in the epidermis, it typically evades a systemic immune response, and is cleared primarily through localised innate and cellular immunity [137]. Severe disease is reported in immunodeficiencies affecting cellular immunity, e.g., AIDS or *DOCK8* deficiency, though not typically CVID [137]. Interestingly, there has been a case of *NFKB2* LOF monogenic CVID with severe MC and associated anti-cytokine antibodies reported [60].

Overall, in a case series of 50 *NFKB2* LOF patients, 32% (16/50) had one or more instances of viral skin infections, including HPV, HSV, VZV, and MCV, with demonstration of dysregulated T-cell and NK function in the population, as well as the production of anti-cytokine antibodies [60]. Certainly, any patient with significant viral skin disease should prompt in-depth genetic and immunological studies.

## 6. Gastrointestinal Viral Infections

Gastrointestinal symptoms are common in CVID, though viral infections are not regarded as a common cause. However, where viral infections are related to gastrointestinal dysregulation in CVID, the effects can be quite profound and may require treatment, stressing the need for consideration and diagnosis in patients with chronic disease. A retrospective study of 132 Finnish patients, 106 probable CVID, and 26 possible CVID, as defined by the European Society for Immunodeficiency/Pan-American Group for Immunodeficiency criteria, reported gastrointestinal manifestations of the condition [138]. The study demonstrated viral colitis caused by norovirus (n = 9), cytomegalovirus (CMV) (n = 1) and HSV (n = 1) [138]. Norovirus was the most common gastrointestinal viral infection found in this cohort, with four of these developing chronic disease [138].

### 6.1. Norovirus

Norovirus is a non-enveloped, positive stranded RNA virus which, in the United States, is responsible for ~21 million cases of acute gastroenteritis annually, and identified in ~60% of all cases with a known infective cause [139]. Worldwide it is said to cause 699 million infections annually, and is responsible for approximately one fifth of all gastroenteritis cases [140]. In most immunocompetent people it is the archetypal “24-h bug”, propagating through faecal-oral transmission, causing symptoms of nausea, diarrhoea, and vomiting for approximately 2–3 days, after an incubation period of around 48 h [139]. A UK study demonstrated an age-adjusted community prevalence of asymptomatic norovirus as 12%, whilst another study showed 47% of symptomatic patients excreted the virus for 21 days or more [141,142]. Indeed, in a study of patients experimentally infected, post-symptomatic viral shedding occurred for a median average of 28 days and up to 8 weeks [143]. This data highlights the high incidence of exposure to the virus in any population, including CVID patients.

In the immunocompetent population, studies have shown strain specific short-term immunity to norovirus lasting around 6 to 24 months, whilst mathematical modelling suggests post infective immunity can last between 4.1 and 8.7 years in the broader population [144,145]. Antibody-mediated immunity seems to play a significant role in this protection, as evidenced by passive immunity conferred by maternal immunoglobulins in breastmilk, the association of immunity and post-vaccine salivary IgA levels, and the correlation of protection and high-titres of blocking IgG antibodies [146,147,148]. Supporting this is the discovery that novel strains of norovirus develop mutations that lead to amino acid changes at recognised antibody binding epitopes of the capsid proteins, suggesting evasion of humoral immunity determines infectivity in those previously exposed [149]. Due to deranged humoral immunity, patients with CVID are therefore particularly susceptible and in some cases shed the virus for years, a condition known as chronic norovirus infection [139].

#### 6.1.1. Norovirus and CVID Enteropathy

Gastrointestinal symptoms are a prominent feature of the CVID phenotype, with one study in an unselected population demonstrating frequent bloating (34%), abdominal pain (30%), and diarrhoea (26%) [150]. Causes for these symptoms range from infective, to an inflammatory bowel disease (IBD)-like phenotype which responds to immunosuppressives, to CVID enteropathy; a coeliac disease mimic, constituting villous atrophy, and malabsorption [139].

Endoscopic and histological abnormalities in CVID patients with GI symptoms are common. In a selected population of 50 CVID patients with gastrointestinal symptoms, high rates of malabsorption (54%) and villous atrophy (51%; 21/41) were observed [151]. In another study, 67% (4/6) of CVID patients with a prior diagnosis of GI disease and 88% (21/24) with no prior diagnosis were found to have abnormalities on endoscopy and histology including inflammation, villous atrophy and nodular lymphoid hyperplasia [152].

CVID enteropathy occurs in ~5% of patients with CVID, with those with defective T-cell function found to be at particular risk [139,151]. Symptoms include diarrhoea and malabsorption which can be quite profound, progressing to steatorrhea, protein-losing enteropathy, and the need for parenteral nutrition [139]. Histology in these patients reveals mild to severe villous atrophy, flattened and vacuolated enterocytes, increased epithelial apoptosis, and chronic inflammatory infiltrates [139]. The presence of lymphoid follicular hyperplasia and the absence of plasma cells can help differentiate CVID enteropathy from coeliac disease, but does not rule out the coexistent diagnosis of coeliac disease [151].

For a long time the cause of CVID enteropathy was unclear, though in 2015, Woodward et al. demonstrated an association with chronic norovirus excretion, proving chronic norovirus infection in all eight of their patients with CVID enteropathy, and an absence of infection in asymptomatic cases [153]. This study demonstrated viral shedding for up to 1200 days in one case, and another who had the same viral strain over the course of illness, suggesting persisting infection, rather than clearance and re-infection [153]. In the three patients who cleared the virus, one spontaneously and two following prolonged ribavirin treatment, there was rapid symptomatic improvement, resolution of intestinal inflammation and restoration of duodenal villi on histology [139,153]. This finding has since been challenged, with one article arguing that chronic norovirus is a symptom and not the cause of CVID enteropathy, though a fundamental issue seems to be a disagreement on the definition of “CVID enteropathy”, with multiple different published criteria allowing for a broad cohort of patients, not all of which are characterised by a coeliac-like disease [154]. Certainly, work is required to clearly define the entity of GI disease relating to chronic norovirus in CVID and establish the causality of the relationship.

A case of chronic norovirus and parechovirus co-infection has since been reported in CVID, with the patient developing villous atrophy, protein losing enteropathy, requiring parenteral nutritional support and not responding to pleconaril [155]. The significance of the parechovirus co-infection is unclear given the known role of norovirus in the development of enteropathy.

Immunologically, the development of villous atrophy in CVID, with and without infection, has been associated with an absence of intestinal IgA+ plasma cells and a mixed interferon type I/III and II signature, which is exaggerated in those with norovirus infection [156]. Chronic norovirus is known to occur in non-CVID patients with absent intestinal B-cells, further highlighting their role in preventing the disease [157]. Other reports have highlighted the large number of T-cells present in the gastrointestinal tract in CVID enteropathy, raising the possibility of an autoimmune manifestation as an indirect result of compensatory immunological mechanisms [155].

#### 6.1.2. Treatment of Chronic Norovirus in CVID

Throughout various reports, ribavirin seems to have the highest rate of sustained clearance of norovirus in these patients, though only in a minority (12.5–29%) of those treated [139,158,159]. Enteral immunoglobulin therapy for norovirus has shown promise in patients with a range of immunodeficiencies, especially post-transplant patients [139,160,161]. However, there is only one successful case reported in CVID, and a case series of two patients receiving the treatment showed no benefit, raising the likelihood of publication bias in an overall inconsistent therapeutic option [139,162,163].The broad-spectrum antiparasitic nitazoxanide was previously shown to treat norovirus in a patient post bone marrow transplant symptomatic for two weeks, and this was further supported by clearance in two of eight (25%) CVID patients, for 6 and 14 months until recurrence in another report [158,164]. However, it has previously been noted that nitazoxanide may inhibit PCR, seen in one report in both the norovirus PCR test and a separate control, suggesting a risk of false negatives and only the illusion of viral clearance [165]. Favipiravir has been shown to improve diarrhoea and reverse weight loss in the condition, whilst one report of interferon alpha-2a indicated non-efficacy [163,166]. Given the weight of evidence showing the severe impact of chronic norovirus and enteropathy in the CVID population, the development and study of reliably effective treatments for chronic norovirus infection is of the utmost importance moving forwards.

### 6.2. Cytomegalovirus Colitis

CMV, also known as HHV-5, is an enveloped dsDNA virus and part of the betaherpesvirinae subfamily [114]. Infection is often asymptomatic, though it can cause a glandular fever-like illness, and can pass to the placenta in pregnant women, causing congenital CMV infection [114]. CMV infection is common, with seroprevalence ranging from 45–100% worldwide, and after the initial infection, the virus becomes latent in multiple cell types, reactivating if the host becomes immunocompromised [167]. CMV colitis in CVID is exceedingly rare but does occur, with 13 reported cases reported in the English literature and 1% (3/252) developing visceral CMV infection in the French prospective study [49,168]. The most common symptoms noted in CVID patients with CMV colitis were worsening, sometimes bloody diarrhoea and weight loss [168,169]. Biopsy and histological analysis is required for diagnosis, with serum viral PCR of limited use [168]. In both immunocompetent and immunocompromised patients, ganciclovir is the antiviral choice for the condition [168,170,171].

In one reported case of a male with CVID diagnosed with CMV colitis, the patient presented with acute gastrointestinal bleeding on a background of known CMV viraemia which had become resistant to ganciclovir, thus requiring foscarnet [168]. Endoscopy showed a bleeding gastrointestinal ulcer, with biopsies demonstrating CMV infection. Due to intolerable side effects to the foscarnet, anti-CMV immunoglobulin (Cytogam^®^) therapy was commenced, with subsequent sustained virological and clinical improvement, though a single case report does not validate this therapeutical approach [168].

CMV colitis is a known complication of IBD, particularly in those on corticosteroids or immunosuppressives [172]. Given that there is a high incidence of IBD in CVID, and CMV colitis can mimic IBD-like disease, it is worth maintaining an index of suspicion for CMV colitis in any CVID patient presenting with IBD-like symptoms, and to consider a superadded CMV infection in any acute exacerbation of IBD, particularly if it is refractory to treatment [151,154]. This is especially important given the range of effective therapies for CMV and its associated high mortality. Regarding treatment, intravenous ganciclovir is considered first line, with oral valganciclovir available for outpatient care, whilst foscarnet, cidofivir, fomivirsen and Cytogam^®^ are also options, especially in the case of drug resistance [168,173].

### 6.3. Herpes Simplex Virus Colitis

HSV colitis is a rare complication, sometimes seen complicating IBD in the context of immunosuppression, though there are fewer than 10 reported cases of this in the English literature [174,175]. Within this cohort, all HSV colitis cases present as acute severe UC, with subsequent colectomies, and sometimes delayed virological diagnosis [175]. There have been previous reports of successful treatment with the antiviral aciclovir [174]. Regarding CVID, one case report details a 69 year old female with chronic diarrhoea relating to rectosigmoidal and caecal ulcerations, found to be HSV positive, with prompt clinical remission following initiation of valaciclovir [176]. There is a high incidence of IBD-like disease in patients with CVID; 2–14% across various studies, whilst in another 40% (14/35) patients with gastrointestinal symptoms had findings consistent with microscopic colitis, Crohn’s disease (CD) or ulcerative colitis (UC) [151,154]. Thus, it is worth maintaining an index of suspicion for HSV colitis in those presenting with acute severe UC or evidence of colonic inflammation.

## 7. Hepatic Viral Infections

Viral hepatitis caused by hepatitis B virus (HBV) and hepatitis C virus (HCV) has been reported in the CVID population [177]. HBV is an enveloped, DNA virus that belongs to the Hepadnaviridae family and HCV is an enveloped +ssRNA virus belonging to the Flaviviridae family, genus *Hepacivirus* [178,179]. Both viruses can lead to chronic infection and are significant risk factors for the development of a cirrhosis, fibrosis, and hepatocellular carcinoma in the normal population. In general, the prevalence of HBV in North America and Western Europe is 0.7–1.6%, and HCV in the same regions is 1.3–2.4% [179,180]. By comparison, a 2008 study of 334 CVID patients across Europe demonstrated a prevalence of HBV (1%) and HCV (6%), indicating the outsized burden of HCV in CVID [22].

CVID itself is not considered pathogenically susceptible to the virus and it has been posited that contamination of blood products has been the main source of infection [177]. Certainly, this is supported by multiple reported outbreaks of HCV relating to contaminated immunoglobulin [181,182,183,184,185]. In two large studies by the same research group following CVID patients, published 12 years apart, there were reported reductions in the overall cumulative incidence of viral hepatitis from 6.5% (combined viral hepatitis) to 1% (HBV) and 1.9% (HCV), possibly indicating the role of better immunoglobulin preparation measures [11,23,177].

Due to the established risk of HCV-contaminated immunoglobulin transfusions, baseline hepatitis screening and subsequent monitoring are advisable for this cohort. A 2002 multicentre study of 1243 patients with PID receiving IgRT showed that only 60% were tested at least once for HCV, whilst in the 71 HCV-infected patients, only 10% spontaneously cleared the virus, 30% proceeded asymptomatically, and 40% developed a rapidly progressive infection, resulting in end-stage liver disease [186]. Of the PIDs studied in this cohort, CVID patients were found to have worse outcomes than those with XLA [186].

There have been varying reports on the impact of HCV in the CVID population, with some indicating an increased rate of morbidity and mortality whilst, in another small study, none of the 15 CVID patients developed severe disease or died from the condition [177,187]. Of the two CVID patients in one report who developed HBV infections, one developed cirrhosis, whilst one recovered [23]. Treatment of HBV and HCV is beyond the scope of this review, other than to note that treatment has improved significantly in recent years, with multiple antivirals available for both, and high cure rates now seen in HCV whilst profound virological suppression is an achievable aim in chronic HBV [178,179].

Regarding unique susceptibilities in monogenic CVID, LOF mutations in ICOS have been associated with mild chronic HHV-6 hepatitis and severe HHV-6 panenteritis who unfortunately died post allogenic haemopoetetic stem cell transplant (HSCT) [188]. Like CMV, HHV-6 is a betaherpesvirus and can be treated with aciclovir, ganciclovir, and foscarnet [113].

## 8. Central Nervous System (CNS) Viral Infections

### 8.1. Viral Meningoencephalitis

Even in immunocompetent hosts, enteroviruses, including echoviruses, coxsackieviruses and polioviruses, cause >80% of viral meningitides with a known cause [189]. Normally these viral infections are brief and self-limiting, with no residual neurological sequelae, however patients with CVID and primary antibody deficiencies in general, are particularly susceptible to enteroviruses, and numerous cases of chronic meningoencephalitis secondary to non-polio enteroviruses have been reported, with significant mortality or residual neurological morbidity (e.g., hydrocephalus, learning disability, quadriplegia) reported [190,191,192]. One such study reported of a three year old with CVID on IgRT, presenting with an echovirus meningoencephalitis, eventually responding to intraventricular immunoglobulin [190]. In another case, a patient with NFKB2-associated CVID died following a period of neurodegenerative decline, with multiple negative serological tests, and was found to have evidence of chronic lymphocytic meningoencephalitis secondary to coxsackie A16 virus at postmortem [193].

The anti-enteroviral pleconaril has been used successfully in the treatment of chronic enteroviral meningoencephalitis caused by echovirus 6, with subsequent viral clearance in the cerebrospinal fluid (CSF) and marked clinical improvement [194]. A literature review of enteroviruses in PIDs prior to use of pleconaril did not identify a consistent benefit from pre-existing acute therapies, including intrathecal immunoglobulin, for chronic meningoencephalitis [191]. Later studies into pleconaril showed 75% (12/16) of patients with chronic enterovirus meningoencephalitis and agammaglobulinemia (CEMA) or hypogammaglobulinaemia improved with treatment [195]. Pleconaril appears to be no longer available outside the clinical trial setting, even for compassionate use, indicating the need for an available treatment for this cohort of patients, especially given the poor outcomes with alternative available therapies [194,196,197].

### 8.2. Other CNS Viral Infections

Other reported viral causes of meningoencephalitis in CVID include CMV, BK virus, JC virus, mumps, West Nile virus (WNV), VZV, adenovirus, and HSV [68,71,190,198,199,200,201,202,203]. Arboviruses, which spread through arthropod vectors (e.g., mosquitoes), are the second most common viral causes of meningoencephalitis, and one such, the flavivirus WNV, has previously been associated with an epidemic in the USA [189]. The first report of WNV-associated meningoencephalitis in a patient with CVID reported the patient making a full recovery following empirical treatment, with no lasting neurological sequelae [200]. Whilst enteroviruses and arboviruses most commonly cause epidemics, HSV-1 is the most common cause of sporadic viral encephalitis in the USA [189]. Only treatment of HSV encephalitis with aciclovir has been adequately studied to be recommended as effective, whilst there is evidence of benefit for aciclovir in VZV encephalitis, aciclovir in suspected undifferentiated viral encephalitis, ganciclovir +/− foscarnet in CMV encephalitis, and aciclovir/ganciclovir for B virus encephalitis [204].

To again highlight the risk of live attenuated vaccines in the CVID population, both vaccine-derived poliovirus and vaccine strain measles inclusion-body encephalitis have been reported in CVID or CVID phenotype patients [205,206]. Overall, these cases indicate the need for prompt serological CSF investigations in patients with CVID.

### 8.3. Vaccine Derived Poliovirus

Poliovirus is a non-enveloped +ssRNA enterovirus, transmitted through the faecal-oral route [207]. Infection is most commonly asymptomatic, though it can result in mild constitutional symptoms, gastroenteritis and, in <1% of cases, paralysis [207,208]. This is due to the cytopathic virus’ ability to infect and destroy the anterior horn cells of the spinal cord, causing flaccid paralysis, although other CNS structures can be infected as well [207,208]. Vaccines were developed in the 1950s, and an inactivated poliovirus vaccine (IPV) and the live attenuated oral poliovirus vaccine (OPV) are currently available. The OPV was instrumental in eradicating 99.9% of worldwide poliovirus infections due to its low cost, high efficacy, and induction of a strong intestinal mucosal immunity [207,208,209]. However, some strains used in the OPV are highly neurovirulent, engendering vaccine-derived polioviruses (VDPV) which can lead to active infection. Within the CVID population, three CVID cases of likely VDPV with long term excretion and no history of clinical symptoms have been reported, whilst there is one report of CVID being unmasked by infection and vaccine-associated paralytic poliomyelitis (VAPP) following the OPV [210,211]. An analysis of all PID related VDPV cases between 1962 and 2016 showed 15% (16/107) were in patients with CVID, with 62.5% (10/16) of these developing VAPP [209]. Compared to other primary antibody deficiencies, CVID patients were the least likely to clear VDPV, likely in part due to the heterogeneity of the disease with some CVID subsets having more marked dysregulated cellular immunity that might explain the difference [209]. A study of patients with PID between 2008–2013 in Bangladesh, China, Iran, Philippines, Russia, Sri Lanka, and Tunisia showed 3% (17/562) had poliovirus detected in their stool, with no recorded progression to neurological disease, demonstrating its ongoing risk [212]. With the introduction of the IPV to every country worldwide since 2019, the incidence of vaccine-associated disease associated with OPV can be expected to lessen [213].

## 9. Ocular Viral Infections

Patients with PID and CVID have been reported to experience ocular complications, including viruses, though no data has been published specifically detailing the experience of those with CVID [214]. A 10 year old with CVID presented with blurred vision and was found to have a cotton wool spot on retinal imaging suggestive of CMV retinitis [173]. The patient had confirmed CMV viraemia, resolving after administration of intravenous ganciclovir [173]. In another report, a 12 year old boy presented with CMV retinitis with retinal detachment, and despite treatment with ganciclovir, the patient’s eyesight after surgery remained impaired [215].

## 10. Oncoviruses

There are seven known oncoviruses—viruses that cause tumours—in humans; Epstein Barr virus (EBV), human papillomaviruses (HPVs), Human Herpesvirus 8 (HHV-8, also known as Kaposi sarcoma-associated herpesvirus), hepatitis B virus (HBV), hepatitis C virus (HCV), human T-lymphotropic virus-1 (HTLV-1), and Merkel cell polyomavirus (MCPyV) [216]. Though these viruses promote tumour development, typically, infection is met with a competent host immune response, with viral clearance and the risk of cancer ameliorated. However, in patients with immunodeficiency, particularly those affecting T and NK cells, these viruses can persist over a period of years to decades, leading eventually to tumourigenesis [216]. CVID is understood to be a predominantly B-cell mediated immunodeficiency with varying degrees of T-cell dysregulation, though malignant and granulomatous manifestations of persisting oncovirus infection, especially EBV and HHV-8, have been reported.

### 10.1. Ebstein Barr Virus

EBV, also known as HHV-4, is an enveloped dsDNA virus belonging to the gammaherpesvirus subfamily [114]. It first enters the host through the airways, spreading to the submucosal lymphoid tissues, infecting naïve B-cells and, if not eliminated by cytotoxic T-cells, eventually progresses to the memory B-cell reservoir, later becoming actively infective again in plasma cells [217]. This process describes primary EBV infection and latency which, if persistent and poorly controlled in the immunocompromised host, can lead to a wide range of cancers including various lymphomas, gastric cancers, and smooth muscle tumours [216]. Persisting viraemia can be identified by measuring EBV viral load and histology samples of these various malignancies often show EBV positivity [217]. Certain monogenic subsets of CVID, such as NF-κB1 deficiency, which is the most common monogenic cause of CVID in Europeans, have been noted to have an increased risk of lymphoproliferation [71]. It has been posited that EBV infection could play a significant role, and indeed, a case of *NFKB1* haploinsufficiency associated with immunodeficiency and EBV-driven lymphoproliferation has been reported [218].

### 10.2. Human Herpesvirus 8 and Granulomatous-Lymphocytic Interstitial Lung Disease

HHV-8 is an enveloped dsDNA virus that has a high tropism for multiple cell types, including lymphocytes, endothelial cells and keratinocytes, and is associated with Kaposi’s sarcoma, primary effusion lymphoma, and multicentric Castleman’s disease [219,220]. The virus has also been implicated in the development of granulomatous-lymphocytic interstitial lung disease (GLILD) in CVID, a severe complication reported in between 8–20% of cases [221,222]. GLILD is a restrictive lung disease presenting with progressive dyspnoea and defined histologically by the presence of non-caseating granuloma and lymphocytic infiltrate in the lung [219,221]. It is often associated with extrapulmonary involvement, including splenomegaly, cytopenias, and lymphoproliferation [223]. Systemic corticosteroids and immunosuppressives, such as azathioprine and mycophenolate mofetil, form the mainstay of treatment for the disease [221]. Regarding the role of HHV-8, one study demonstrated a significantly higher prevalence of HHV-8 infection in 9 CVID patients with GLILD (66%) compared to 21 controls (5%) [224]. Though this relationship exists, the exact mechanism by which HHV-8 may drive granulomatous disease in the development of GLILD is unclear. It has been proposed that the interplay between HHV-8 infection and CVID immunodeficiency drive a dysregulated cytokine response, and that elevated TNF-alpha and interleukin (IL)-6 in particular may then promote the formation of granulomas [224,225]. Indeed, these specific cytokines are known to be elevated in HHV-8 induced cancers [226]. In any case, the causal relationship remains ill-defined and represents an avenue for research in a disease which is, as yet, poorly understood and sub-optimally managed.

## 11. Other Viruses

### 11.1. Human Immunodeficiency Virus (HIV)

The human immunodeficiency viruses (HIV)-1 and HIV-2 are two species of an enveloped +ssRNA lentivirus, within the family Retroviridae, which insert a DNA copy of the viral RNA genome into the host DNA, for it to be transcribed and translated by the cell to produce viral copies [227]. HIV attaches to CD4+ cells; classically Th cells, though also macrophages; DCs and astrocytes, resulting in the elimination of Th cells through various means and their gradual decline [227]. Infection can be followed with a seroconversion illness after 3–6 weeks, followed by a prolonged asymptomatic phase and then the development of acquired immunodeficiency syndrome (AIDS) [227]. A search through literature yielded a small number of significant reports of HIV infection in CVID, especially pertaining to accurate diagnosis and some interesting pathogenesis. There has never been a reported case of HIV transmitted through IgRT [227].

One case report highlighted a patient with CVID on IgRT, who possibly suffered an acute retroviral syndrome, though seemingly did not undergo seroconversion, ostensibly due to the primary hypogammaglobulinaemia [228]. Despite confirmed exposure to HIV and increasing viral loads, the patient had consistently negative ELISAs (enzyme-linked immunosorbent assay) [228]. Though other case reports highlight positive HIV ELISA tests in patients with CVID, this report indicates the need to keep an open mind about the possibility of false negatives due to an absent humoral response to the virus [229].

Remarkably, there have been four reported cases of spontaneous recovery of immunoglobulin levels in patients with CVID infected with HIV [229,230,231,232]. One patient with a negative HIV test in 1974 and 1980 became positive from 1982 onwards and this was associated with increased immunoglobulins (IgG, IgA, IgM) production to normal levels, as well as the demonstration of specific in vivo antibody responses to tetanus and the production of antibodies to CMV and EBV, with associated improved clinical status [232]. Another case showed a rise in IgM (0.19 g/L to 0.9 g/L) with no mention of IgG whilst on IgRT, whilst a third reported marked IgG (50 g/L) and IgA (5 g/L) hypergammaglobulinaemia, though *E. coli* antigen responses and IgA levels remained abnormal [229,231].

Most recently, and in the first reported case to discuss the role of highly active antiretroviral therapy (HAART), a male diagnosed with CVID treated with IgRT was later noted to have raised IgG (43.9 g/L) and IgM (1.3 g/L) with persistent low IgA and IgE levels, and HIV was subsequently diagnosed. His IgRT was stopped and HAART eventually initiated, with his HIV viral load falling from 472,000 to <50 copies/mL and normalisation of his CD4 count [230]. Functional antibody levels to tetanus toxin and pneumococcal capsular polysaccharides subsequently varied though were intermittently normal. Even with the low viral load on HAART, IgG, and IgM levels remained normal and the patient remained off IgRT and clinically well for six years at the time of publication [230]. It was suggested that this may be due to HIV persisting in both the Th cells and follicular DCs of the germinal centres, driving immunoglobulin production through an unclear mechanism [230]. It is worth noting there have been cases of patients with CVID and HIV infection who did not have normalisation of their immunoglobulins, though in these cases, they were known to be receiving immunoglobulin with anti-HIV antibodies present [231,233]. No explanation for the reversal of CVID hypogammaglobulinaemia was proposed, though AIDS is known to cause hypergammaglobulinaemia, and the possibility of a mechanism which bypasses an inhibitory process in CVID, or a subset of CVID, certainly indicates the need for further research.

### 11.2. Human Parvovirus B19

Human *Parvovirus* B19 (HPV-B19) is a non-enveloped ssDNA virus with a high tropism to erythroid progenitor cells, which can lead to temporary bone marrow infection and transient erythropoiesis arrest [234]. In immunocompetent people, it typically causes erythema infectiosum in children (aka “slapped cheek syndrome”) and, in adults, can cause an acute symmetric polyarthropathy [234]. In immunocompromised hosts, persistent infection may lead to pure red cell aplasia and chronic anaemia [235]. There are two reported cases of patients with CVID presenting with erythroid aplasia secondary to HPV-B19 with resolution following IgRT [235,236]. There was a case of 48 year old male with CVID, presenting with a chronic monoarthritis of the wrist found to have parvoviral B19 DNA in synovial tissues which resolved after initiation of IgRT [237]. In another case, a 6-year old presented with one month of fevers and a polyarticular arthritis and was found to have a parvovirus viraemia as well immunological findings indicative of CVID [238]. The introduction of IgRT resolved the viraemia and constitutional symptoms over 15 months, though the arthritis persisted [238]. Regarding diagnosis of the virus in CVID, HPV-B19 serology can be negative due to dysregulated antibody production and HPV-B19 PCR analysis may be necessary for diagnosis [235]. In all these cases, the initiation of IgRT was sufficient to clear the virus, indicating the importance of considering HPV-B19 infection and immunodeficiency in those presenting with reticulocytopaenic anaemia, and the role of immunoglobulin in treating the syndrome.

## 12. Conclusions

This review clearly highlights the heightened susceptibility of individuals with CVID to viral infections. These can range from self-limiting respiratory viruses to more atypical or severe infections. The inability of the immune system to effectively eliminate these viruses can result in immunodysregulation, prolonged illness, repeated hospitalizations, and death. This underscores the urgency of researching and developing strategies to risk stratify CVID patients, develop an understanding of when to screen for viral infections and determine the most appropriate antivirals for prophylactic and therapeutic use.

The COVID-19 global pandemic was the largest pandemic seen in almost a hundred years. There is an ongoing risk of further global outbreaks, or new deadly strains of SARS-CoV-2, and this data demonstrates the risk of dire outcomes in CVID, with significantly higher rates of morbidity and mortality reported in this patient cohort. Nonetheless, the data shows that vaccination can be effective in around two thirds of CVID patients, with multiple vaccinations conferring added benefit. Furthermore, the presence of SARS-CoV-2 antibodies in replacement immunoglobulin preparations, and efficacy of antivirals in this population highlight the importance of regular treatment and monitoring. Thus, there is an urgent need for specialist societies to recommend preventative and therapeutic strategies, for future SARS-CoV-2 strains, or other pandemics. Thankfully outcomes from COVID-19 are improving in CVID, likely due to a combination of factors, though they remain worse than the general population.

Although the CVID population was one of the most badly affected by the COVID-19 pandemic, there are several ways the cohort may stand to benefit from the subsequent lessons learnt and ensuing scientific endeavours. Firstly, the pandemic demonstrated the benefits of effective antivirals in reducing the severity of illness in CVID, highlighting the potential benefit of novel antivirals for other infections. Thankfully, the pandemic has prompted a surge in novel antiviral research and effort should be made to trial these in CVID patients and make them widely available if effective [239]. In a similar vein, the pandemic demonstrated the efficacy of monoclonal antibody therapies for COVID-19. Though this was unsustainable due to the relatively rapid emergence of antigen escape through evolving SARS-CoV-2 sub-variants, it demonstrated an effective proof of concept [95,240,241]. This therapeutic modality may well provide benefit to other viral infections, especially in patients with low to absent humoral immunity, such as patients with CVID. Multiple monoclonal therapies are in development currently, including for RSV and influenza, and could conceivably benefit CVID patients in future [242]. Finally, despite being known as a condition of aberrant humoral immunity, multiple studies demonstrated the significant benefit of SARS-CoV-2 vaccinations in patients with CVID, with vaccines inducing both humoral and cellular responses, indicating a prophylactic role of vaccination for other viral infections in CVID that requires further exploration.

The study of CVID and its relationship with viral infections underscores the critical importance of the intricate interplay between the immune system and pathogens. The spectrum of immunophenotypes in CVID provide insight beyond just the effect of dysregulated humoral immunity on viral infections, illustrating how varying degrees of impaired cellular immunity can create specific vulnerabilities. Monogenic CVID, particularly *NFKB1*, *NFKB2*, *IKZF1*, *TNFSF12* and *ICOS* mutations, are all reported in viral illnesses, and should be investigated for in severe cases, particularly if there are other associated factors, such as the lymphoproliferative disease seen in *NFKB1* LOF. Certainly, this review has highlighted the need to investigate for monogenic CVID in the context of significant viral skin disease (see Table 2).

Increasingly sophisticated genetic and immunological analytical methods will likely lead to the identification of further monogenic causes of CVID, offering insight into other viral susceptibilities and the greater scope for classification, risk stratification, and targeted therapeutic approaches. Furthermore, the resolution of humoral deficiencies in HIV infection hint at new discoveries at the interface of viral infection and immunological pathogenesis, which, as we progress into an era of gene therapy, may provide new avenues for novel therapeutic approaches [243].

As well as infection, a prominent feature of CVID is autoimmune, lymphoproliferative, and granulomatous disease. As in the case of norovirus and CVID enteropathy, HHV-8 and GLILD or EBV and lymphoma, there is a growing understanding of the aetiological role of viruses within these clinical sequelae. Certainly, studies and characterisation of the role between virus and non-infectious complication in CVID are needed, both to optimise prevention and management of these conditions, as well as elucidate any further similar relationships.

## Figures and Tables

**Table 1:** Revised ESID (2014) diagnostic criteria for CVID.

At least one of the following: ∙ Increased susceptibility to infection ∙ Autoimmune manifestations ∙ Granulomatous disease ∙ Unexplained polyclonal lymphoproliferation ∙ Affected family member with antibody deficiency
AND marked decrease of IgG and marked decrease of IgA with or without low IgM levels (measured at least twice; <2 SD of the normal levels for their age);
AND at least one of the following: ∙ Poor antibody response to vaccines (and/or absent isohemagglutinins); i.e., absence of protective levels despite vaccination where defined ∙ Low switched memory B cells (<70% of age-related normal value)
AND Secondary causes of hypogammaglobulinaemia have been excluded.
AND Diagnosis is established after the fourth year of life (symptoms may be present before)
AND No evidence of profound T-cell deficiency, defined as two out of the following (y = year of life): ∙ CD4 numbers/microliter: 2–6 y < 300, 6–12 y < 250, >12 y < 200 ∙ % Naïve CD4: 2–6 y < 25%, 6–16 y < 20%, >16 y < 10% ∙ T-cell proliferation absent

Adapted from Ameratunga R, Brewerton M, Slade C, et al. Comparison of diagnostic criteria for Common Variable Immunodeficiency Disorder. *Front Immunol.* **2014**, *5*, 105842 [7].

**Table 2:** Summary of Viral Infections Reported in CVID.

Organ System	Viral Infections Reported	Unique Infective Features	Recommended/Reported Treatments	Reported Monogenic CVID Cases
**Respiratory**	SARS-CoV-2 (COVID-19)		Paxlovid^®^ (irmatrelvir/ritonavir)/remdesivir/molnupiravir Prophylaxis: IgRT & vaccination	*NFKB2* LOF [93]
	Rhinovirus (*most common*) [67]	Recurrent and persistent infection Often asymptomatic	Nil	
	RSV	Severe bronchiolitis in infants		*IKZF1* [57]
	Adenovirus	Pneumonia in infant	Cidofivir	
	CMV	Pneumonitis	Ganciclovir	*NFKB2* LOF [69]
	RSV, picornavirus, parainfluenza 1 & 2, influenza, coronavirus, metapneumovirus, enterovirus		Nil	
**Mucocutaneous**	HPV	Severe warts	IgRT	*NFKB2* LOF [69] *TNFSF12* (TWEAK) LOF [58]
	HSV	Chronic intraoral ulcer	Aciclovir/Famciclovir/Valaciclovir Foscarnet Pritelivir/Amenamevir	
		Recurrent HSV		*IKZF1* [56]
		Herpes Vegetans		*NFKB2* LOF [120]
	VZV	Recurrent/severe shingles Severe chickenpox Haemorrhagic vesicles	Aciclovir Prophylaxis: Consider inactivated vaccine (Shingrix^®^)	*NFKB2 LOF* [60] *NFKB2/AIRE* [134]
	MCV	Severe molluscum contagiosum	Cidofivir (if severe) [137]	*NFKB2* LOF [60]
**Gastrointestinal**	Norovirus	Chronic infection CVID Enteropathy	Ribavirin Oral immunglobulin Nitazoxanide Favipiravir	
	CMV	Bloody diarrhoea Weight loss	Ganciclovir Valganciclovir Foscarnet anti-CMV Ig (Cytogam^®^)	
	HSV	Acute severe UC	Aciclovir Valaciclovir	
	HHV-6	Severe enteritis Mild chronic hepatitis	Aciclovir Ganciclovir Foscarnet	*ICOS* LOF [188]
**Hepatic**				
	HBV		Nucleos(t)ide analogues (NUCs) [179] Interferon therapy [179]	
	HCV		Direct-acting antiviral drugs (DAA’s) [178] Pegylated interferon alfa and ribavirin (PR) [178]	
**Central Nervous System**	Vaccine-derived polioviruse	Vaccine-associated paralytic poliomyelitis (VAPP)	Nil	
	Coxsackie A16	Chronic lymphocytic mengingoencepahilitis	Nil	*NFKB2* LOF [193]
	SARS-CoV-2	Acute cerebellitis and myeloradiculitis	Convalescent plasma, steroids and IVIg	
	CMV	Meningoencephalitis	Ganciclovir +/− Foscarnet	
	HSV		Aciclovir	
	VZV			
	JC virus			*NFKB1* LOF [71]
	Echovirus		Intraventricular immunoglobulin Pleconaril	
	BK virus, mumps, West Nile virus, adenovirus.		Nil	
**Ocular Viruses**	CMV	Retinitis Retinal detachment	Ganciclovir	
**Oncoviruses**	EBV	Lymphoproliferation	Nil	*NFKB1* LOF [218]
	HHV-8	Associated with GLILD	Nil	
**Other**	HIV	Normalisation of Ig levels	HAART	
	Parvovirus (HPV-B19)	Reticulocytopenic anaemia Chronic monoarthritis	IgRT	

CVID, common variable immunodeficiency; IgRT, immunoglobulin replacement therapy; CMV, cytomegalovirus; Ig, Immunoglobulin; RSV, respiratory syncytial virus; HPV, human papillomavirus; HSV, herpes simplex virus; VZV, varicella zoster virus; MCV, molluscum contagiosum virus; HHV, human herpesvirus; HBV, hepatitis B virus; HCV, hepatitis C virus; IVIg, intravenous immunoglobulin; EBV, Epstein Barr virus; GLILD, granulomatous-lymphocytic interstitial lung disease; HIV, human immunodeficiency virus; HAART, highly active antiretroviral therapy; HPV-B19, Human Parvovirus B19.
